# Supplementary material for: Viral Metagenomics Reveals Diverse Viruses in Tissue Samples of Diseased Pigs
Source: Viruses. 2022 Sep 15;14(9):2048. doi: 10.3390/v14092048 (PMC9500892; doi:10.3390/v14092048)
Supplement: Supplementary file 1 [file viruses-14-02048-s001.zip › Supplementary Table S1.pdf]

**Sample number**	**Types of tissue samples**	**Sampling location**	**Pig age**	**Clinical symptoms**	**Whether the result of traditonal method match with viral metagenomics or not**
Pig01	Liver、Lung、Lymph node	Anqing	Nursery	Respiratory symptoms	N
Pig02	Liver、Spleen、Kidney	Huainan	Nursery	Respiratory symptoms	N
Pig03	Liver、Spleen、Lymph node	Hefei	Nursery	Respiratory symptoms	Y
Pig04	Lung、Lymph node	Hefei	Nursery	Respiratory symptoms	N
Pig05	Lung	Hefei	Stillbirth	Reproductive disorders	N
Pig06	Liver、Lymph node	Hefei	Stillbirth	Reproductive disorders	N
Pig07	Liver、Kidney	Liuan	Finishing	Respiratory symptoms	N
Pig08	Spleen、Lung	Huangshan	Nursery	Respiratory symptoms	Y
Pig09	Liver、Lymph node	Fuyang	Nursery	Respiratory symptoms	N
Pig10	Liver、Spleen、Lung	Wannan	Nursery	Respiratory symptoms	N
Pig11	Liver、Spleen、Lung、Kidney、Lymph node	Fuyang	Nursery	Respiratory symptoms	N
Pig12	Liver、Spleen、Lung	Hefei	Stillbirth	Reproductive disorders	Y
Pig13	Liver、Spleen	Hefei	Stillbirth	Reproductive disorders	N
Pig14	Liver、Lung	Hefei	Nursery	Respiratory symptoms	data missing
Pig15	Lung	Huaibei	suckling	Respiratory symptoms	data missing
Pig18	Spleen、Lung	Fuyang	Stillbirth	Reproductive disorders	N
Pig21	Lung、Kidney	Huaibei	suckling	Reproductive disorders	Y
Pig22	Liver、Spleen、Lung	Fuyang	Nursery	Acute death	data missing
Pig23	Spleen、Lung	Fuyang	Nursery	Acute death	data missing
Pig24	Liver、Spleen	Hefei	Nursery	Diarrhea	N
Pig25	Liver、Lung	Hefei	Nursery	Diarrhea	N
Pig26	Lung	Huaibei	suckling	Respiratory symptoms	data missing
Pig27	Liver、Spleen、Lung	Chuzhou	Finishing	Diarrhea	Y
Pig28	Liver、Spleen、Lung、Kidney	Chuzhou	Finishing	Diarrhea	Y
Pig29	Liver、Spleen、Lung、Kidney、Lymph node、Brain	Chuzhou	Finishing	Diarrhea	Y
Pig30	Liver、Spleen、Lung、Kidney	Bengbu	Nursery	Diarrhea、Respiratory symptoms	Y
Pig31	Liver、Spleen、Kidney	Chuzhou	Nursery	Diarrhea	Y
Pig32	Liver、Spleen、Lung、Kidney	Chuzhou	Nursery	Diarrhea	Y
Pig33	Liver、Lung	Liuan	suckling	Diarrhea	Y
Pig34	Liver、Lung	Liuan	suckling	Diarrhea	Y
Pig35	Liver、Spleen、Lung、Kidney	Hefei	Nursery	Respiratory symptoms	Y
Pig36	Liver、Spleen、Lung、Kidney	Liuan	Nursery	Diarrhea	Y
Pig37	Liver、Spleen、Lung、Kidney、Lymph node	Liuan	Nursery	Diarrhea	Y
Pig38	Liver、Lung、Kidney	Liuan	Nursery	Diarrhea	N
Pig39	Liver、Spleen、Lung、Kidney、Lymph node	Huaibei	suckling	Diarrhea	Y

Pig40	Spleen、Kidney	Huaibei	suckling	Diarrhea	Y
Pig41	Liver、Spleen、Lung、Kidney、Lymph node	Huaibei	suckling	Diarrhea	Y
Pig42	Liver、Spleen、Lung、Lymph node	Huaibei	suckling	Diarrhea	Y
Pig43	Liver、Spleen、Lung、Kidney	Huai'an (Jiangsu)	Nursery	Lose weight	data missing
Pig44	Liver、Spleen、Lung、Kidney	Huai'an (Jiangsu)	Nursery	Lose weight	data missing
Pig45	Liver、Spleen、Lung、Kidney、Lymph node	Huai'an (Jiangsu)	Nursery	Lose weight	data missing
Pig46	Liver、Spleen、Lung、Kidney	Huai'an (Jiangsu)	Nursery	Lose weight	data missing
Pig47	Liver、Spleen、Lung、Kidney	Huai'an (Jiangsu)	Nursery	Lose weight	data missing
Pig48	Liver、Spleen、Kidney	Huai'an (Jiangsu)	Nursery	Lose weight	data missing
Pig49	Liver、Spleen、Lung、Kidney、Lymph node	Chengdu (Sichuan)	Nursery	Diarrhea	Y
Pig50	Liver、Spleen、Lung、Kidney	Chengdu (Sichuan)	Nursery	Diarrhea	Y
Pig51	Liver、Spleen、Lung、	Chengdu (Sichuan)	Nursery	Diarrhea	Y
Pig52	Liver、Spleen、Lung、Kidney	Hefei	Nursery	Respiratory symptoms	Y
Pig53	Liver、Spleen、Lung、Kidney、Brain	Hefei	Nursery	Respiratory symptoms	Y
Pig54	Liver、Spleen、Lung、	Hefei	Nursery	Diarrhea	Y
Pig55	Liver、Spleen、Lung、Kidney	Hefei	Nursery	Diarrhea	Y
Pig56	Liver、Lung	Hefei	Stillbirth	Reproductive disorders	Y
Pig57	Liver、Spleen	Hefei	Stillbirth	Reproductive disorders	Y
Pig58	Liver、Spleen、Lung	Hefei	Stillbirth	Reproductive disorders	Y
Pig59	Liver、Lung	Hefei	Stillbirth	Reproductive disorders	Y
Pig60	Liver、Spleen、Lung、Kidney、Brain	Fuyang	Nursery	High fever	Y
Pig61	Liver、Spleen、Lung、Kidney、Brain	Fuyang	Nursery	High fever	Y
Pig62	Liver、Spleen、Lung	Fuyang	Nursery	Diarrhea	Y
Pig63	Liver、Spleen、Lung	Fuyang	Nursery	Diarrhea	Y
Pig64	Liver、Spleen、Lung、Kidney	Suzhou	Nursery	Diarrhea	N
Pig65	Liver、Spleen、Lung、Kidney	Suzhou	Nursery	Diarrhea	N
Pig66	Liver、Spleen、Lung、Kidney	Suzhou	Nursery	Diarrhea	N
Pig67	Liver、Spleen、Lung、Kidney	Bengbu	suckling	Diarrhea	N
Pig68	Liver、Spleen、Lung、Kidney、Lymph node	Bozhou	Nursery	High fever、Lose weight	Y
Pig69	Liver、Spleen、Lung、Kidney、Lymph node	Hefei	Nursery	Lose weight	N
Pig70	Liver、Spleen、Lung	Fuyang	Nursery	Diarrhea	Y
Pig71	Liver、Spleen、Lung、Kidney	Fuyang	Nursery	Diarrhea	Y
Pig72	Liver、Spleen、Lung、Kidney	Fuyang	Nursery	Diarrhea	Y
Pig73	Lung	Anqing	Nursery	Respiratory symptoms	data missing
Pig74	Lung	Anqing	Nursery	Respiratory symptoms	data missing
Pig75	Lung	Anqing	Nursery	Respiratory symptoms	data missing
Pig76	Liver、Spleen、Lung、Kidney、Lymph node	Chuzhou	Unknown	Unknown	data missing
Pig77	Liver、Spleen	Chuzhou	Finishing	Acute death	N
Pig78	Liver、Spleen	Chuzhou	Finishing	Acute death	N
Pig79	Liver、Spleen	Chuzhou	Finishing	Acute death	N
Pig80	Liver、Spleen、Lung、Kidney	Bozhou	Nursery	Diarrhea	Y
Pig81	Liver、Spleen、Lung、Brain	Bozhou	Nursery	Diarrhea	Y

Pig82	Liver、Spleen、Lung、Brain	Bozhou	Nursery	Diarrhea	Y
Pig83	Liver、Spleen、Lung、Kidney	Hefei	suckling	Diarrhea	data missing
Pig84	Liver、Spleen、Lung、Kidney、Brain	Hefei	Finishing	Neurological and respiratory symptoms	N
Pig85	Liver、Spleen、Lung、Brain	Hefei	Finishing	Neurological and respiratory symptoms	Y
Pig86	Liver、Spleen、Lung	Liuan	Nursery	Respiratory symptoms	N
Pig87	Liver、Spleen、Lung	Liuan	Nursery	Respiratory symptoms	N
Pig88	Liver、Spleen、Lung、Kidney、Lymph node	hefei	Nursery	Respiratory symptoms	data missing
Pig89	Liver、Spleen、Lung、Kidney、Lymph node	hefei	Nursery	Respiratory symptoms	data missing
Pig90	Liver、Spleen、Lung、Kidney	Wuhu	Nursery	Respiratory symptoms	data missing
Pig91	Liver、Spleen、Lung、Kidney、Lymph node	Hefei	Nursery	Respiratory symptoms	data missing
Pig92	Liver、Spleen、Lung、Lymph node	Anqing	Finishing	Respiratory symptoms	Y
Pig93	Liver、Spleen、Lung、Kidney、Lymph node	Hefei	Nursery	Diarrhea	Y
Pig94	Liver、Spleen、Lung、Kidney、Lymph node	Hefei	Nursery	Diarrhea	Y
