# Supplementary material for: Viral Metagenomics Reveals Diverse Viruses in Tissue Samples of Diseased Pigs
Source: Viruses. 2022 Sep 15;14(9):2048. doi: 10.3390/v14092048 (PMC9500892; doi:10.3390/v14092048)
Supplement: Supplementary file 1 [file viruses-14-02048-s001.zip › Supplementary Table S2.pdf]

Library number	Library accession	Total reads	Viral reads with E value<10^{-5}	Obtained complete genome	GenBank Accession no.
Pig01	SRR14099162	25, 925	2, 476		
Pig02	SRR14099163	9, 026	131		
Pig03	SRR14099194	72, 306	55		
Pig04	SRR14099195	9, 408	428		
Pig05	SRR14099196	50, 152	25, 466	Parvovirus 2	MW853943
Pig06	SRR14099197	92, 265	1, 948		
Pig07	SRR14099198	62, 275	1, 342	Parvovirus 2	MW853944
Pig08	SRR14099199	11, 248	1, 120	Circoviridae	MW853933
Pig09	SRR14099212	41, 103	2, 240	Circoviridae	MW853934
Pig10	SRR14099213	10, 229	262		
Pig11	SRR14099214	7, 642	268		
Pig12	SRR14099229	54, 348	2, 656		
Pig13	SRR14099237	97, 956	6, 706		
Pig14	SRR14099238	54, 217	8, 761	Circoviridae, Parvovirus 6	MW853935, MW853954
Pig15	SRR14099239	58, 098	822		
Pig18	SRR14099248	6, 634	1, 583		
Pig21	SRR14099249	117, 712	1, 175		
Pig22	SRR14099471	7, 113	239		
Pig23	SRR14099472	7, 732	101		
Pig24	SRR14099473	226, 205	8, 180		
Pig25	SRR14099474	208, 211	13, 876		
Pig26	SRR14099971	257, 651	2, 849	Parvovirus 2	MW853945
Pig27	SRR14100086	210, 484	4, 743	Circoviridae	MW853936
Pig28	SRR14100087	111, 997	7, 664	Arteriviridae, Flaviviridae	MW853923, MW853924
Pig29	SRR14100100	71, 420	819		
Pig30	SRR14100583	53, 803	2, 853	Parvovirus 5	MW853951
Pig31	SRR14100584	87, 896	791		
Pig32	SRR14100585	101, 411	4, 167	Flaviviridae	MW853925
Pig33	SRR14100594	24, 930	109		
Pig34	SRR14100603	96, 612	2, 861		
Pig35	SRR14101505	89, 816	410		
Pig36	SRR14101506	85, 538	5, 249	Flaviviridae, Parvovirus 2	MW853926, MW853946
Pig37	SRR14101520	197, 595	16, 077	Flaviviridae	MW853927
Pig38	SRR14101522	115, 079	1, 116		

Pig39	SRR14101703	573, 245	7, 672	Flaviviridae	MW853928
Pig40	SRR14126926	147, 632	4, 781		
Pig41	SRR14126927	100, 650	1, 125		
Pig42	SRR14126929	152, 218	7, 372	Flaviviridae	MW853929
Pig43	SRR14126930	184, 502	3, 240	Parvovirus 2	MW853947
Pig44	SRR14126931	273, 946	8, 971		
Pig45	SRR14126932	192, 211	981		
Pig46	SRR14126933	109, 264	6, 307		
Pig47	SRR14126934	219, 303	2, 943		
Pig48	SRR14127098	194, 685	7, 990	Circoviridae, Parvovirus 2	MW853937, MW853948
Pig49	SRR14127099	70, 375	17, 953	Circoviridae	MW853938
Pig50	SRR14127129	542, 732	3, 886	Circoviridae	MW853939
Pig51	SRR14127130	183, 288	20, 570	Circoviridae	MW853940
Pig52	SRR14127132	126, 993	5, 029		
Pig53	SRR14127133	124, 225	1, 586		
Pig54	SRR14127134	66, 832	292		
Pig55	SRR14127135	145, 583	1, 309		
Pig56	SRR14127136	77, 627	3, 257		
Pig57	SRR14127173	620, 706	1, 988		
Pig58	SRR14127174	243, 608	11, 171		
Pig59	SRR14127175	76, 622	1, 467		
Pig60	SRR14127194	410, 072	8, 109	Parvovirus 7	MW853958
Pig61	SRR14127273	526, 967	8, 523		
Pig62	SRR14127285	129, 425	3, 273	Flaviviridae	MW853930
Pig63	SRR14127288	89, 088	4, 194	Flaviviridae	MW853931
Pig64	SRR14127289	64, 789	2, 087		
Pig65	SRR14127290	110, 910	1, 102		
Pig66	SRR14127291	106, 516	4, 233		
Pig67	SRR14127292	377, 270	1, 539		
Pig68	SRR14127294	158, 203	11, 428	Parvovirus 2	MW853949
Pig69	SRR14127307	86, 201	799		
Pig70	SRR14127350	192, 626	12, 554		
Pig71	SRR14127351	355, 671	7, 437		
Pig72	SRR14127364	102, 321	4, 408	Parvovirus 3	MW853950
Pig73	SRR14127384	547, 152	34, 021	Parvovirus 6, Parvovirus 7	MW853955, MW853959

Pig74	SRR14127385	98, 843	1, 266	Parvovirus 7	MW853960
Pig75	SRR14127423	633, 748	196, 045	Parvovirus 6, Parvovirus 7	MW853956, MW853961
Pig76	SRR14127424	73, 949	2, 538		
Pig77	SRR14127425	118, 950	1, 590		
Pig78	SRR14127426	26, 645	1, 428		
Pig79	SRR14127434	83, 958	990		
Pig80	SRR14127435	118, 229	5, 245		
Pig81	SRR14127459	471, 375	1, 934		
Pig82	SRR14127475	45, 077	2, 661		
Pig83	SRR14127483	417, 417	8, 534		
Pig84	SRR14127484	788, 060	31, 307		
Pig85	SRR14127534	615, 846	57, 355	Circoviridae, Parvovirus 5, Parvovirus 6	MW853941, MW853952, MW853957
Pig86	SRR14127578	275, 809	8, 592	Circoviridae, Parvovirus 5	MW853942, MW853953
Pig87	SRR14127579	218, 507	4, 013		
Pig88	SRR14127581	79, 864	2, 892		
Pig89	SRR14127586	82, 527	957		
Pig90	SRR14127601	1, 702, 271	1, 187, 870		
Pig91	SRR14127782	111, 474	1, 152		
Pig92	SRR14127784	67, 119	3, 344	Parvovirus 7	MW853962
Pig93	SRR14127787	124, 774	2, 315	Flaviviridae	MW853932
Pig94	SRR14127791	143, 366	8, 892		
