# Supplementary figures and images for: Viral Metagenomics Reveals Diverse Viruses in Tissue Samples of Diseased Pigs
Source: Viruses. 2022 Sep 15;14(9):2048. doi: 10.3390/v14092048 (PMC9500892; doi:10.3390/v14092048)

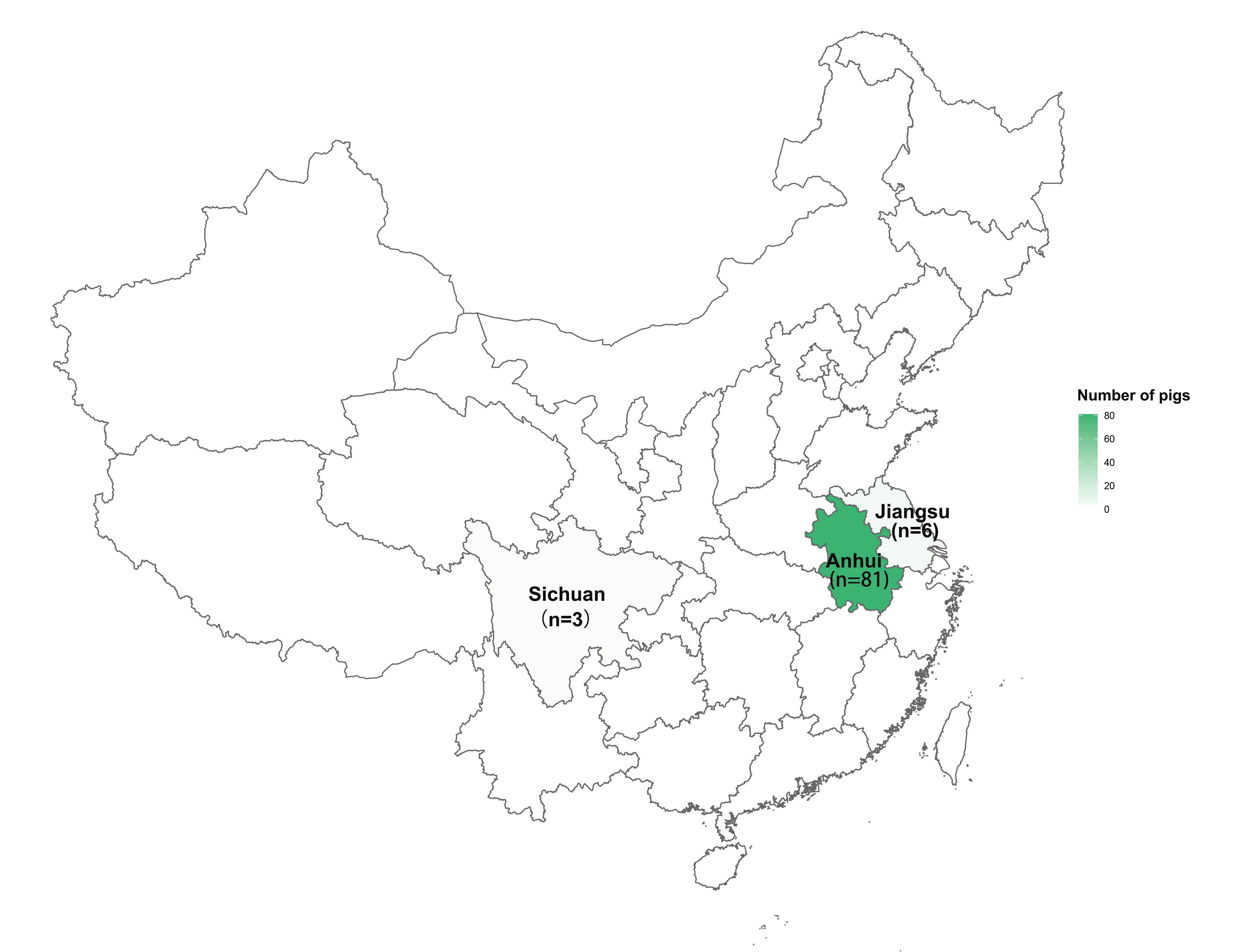

Supplement: Supplementary file 1 [file viruses-14-02048-s001.zip › Supplementary Figure S1.jpg]

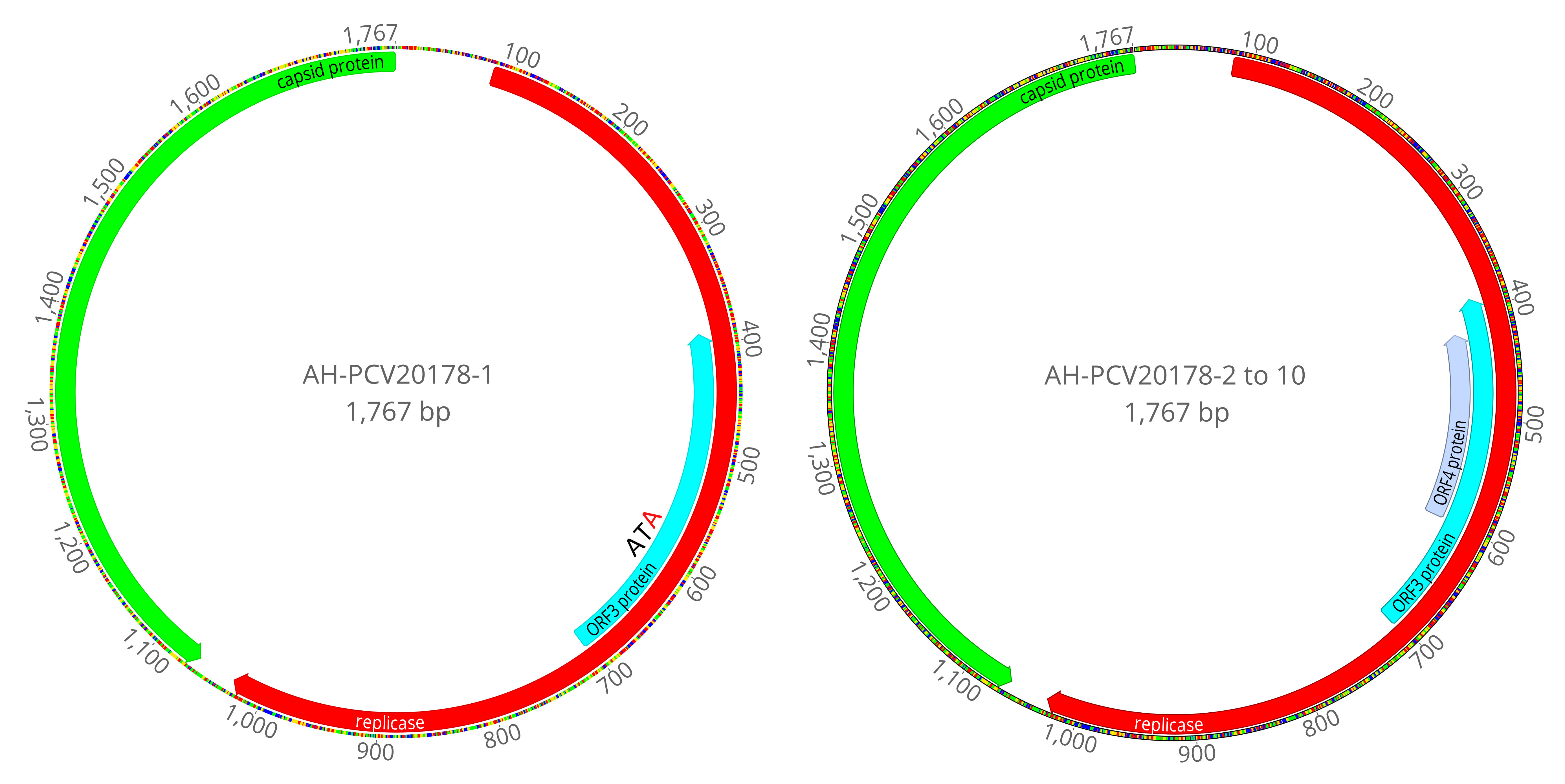

Supplement: Supplementary file 1 [file viruses-14-02048-s001.zip › Supplementary Figure S2.jpg]

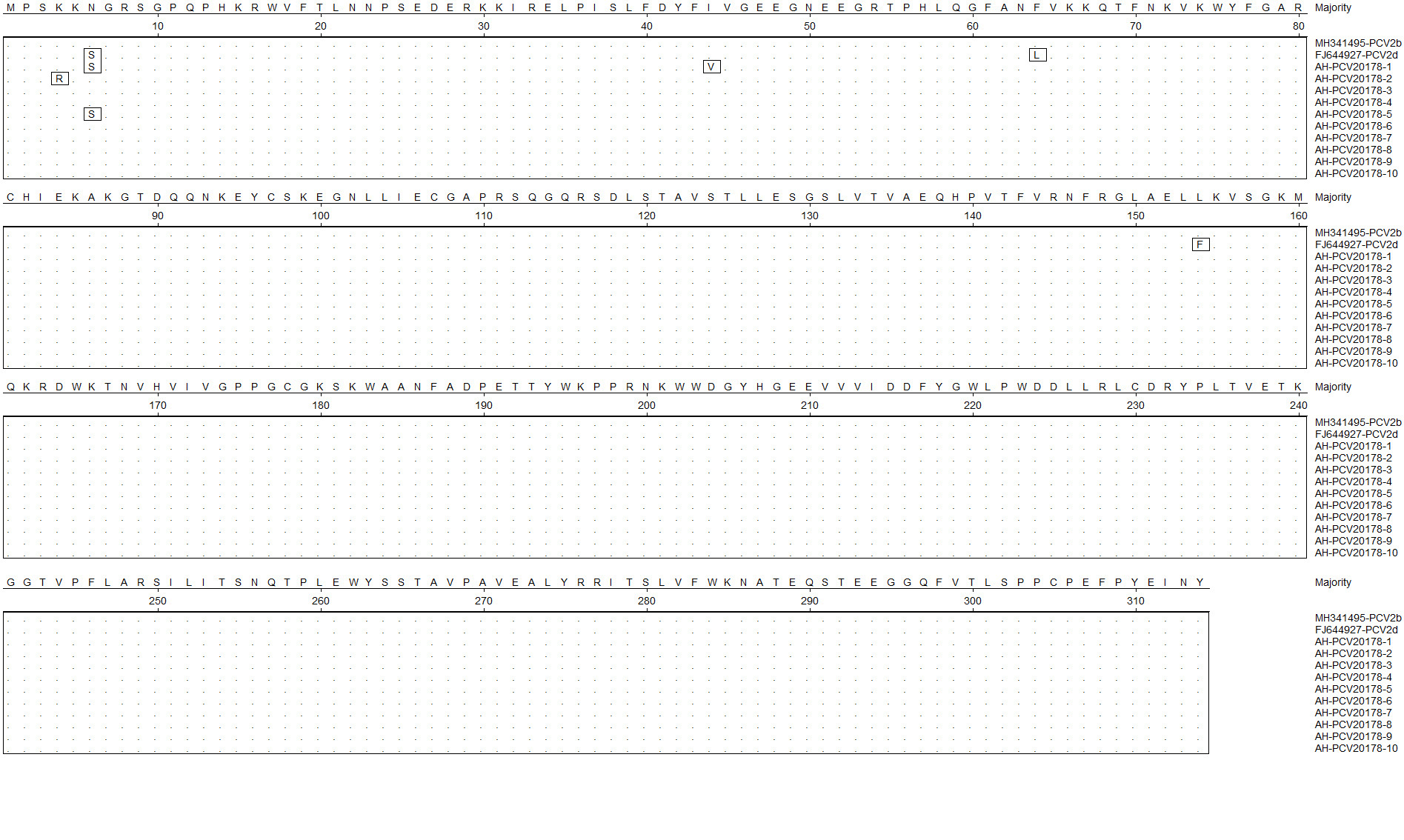

Supplement: Supplementary file 1 [file viruses-14-02048-s001.zip › Supplementary Figure S3.jpg]

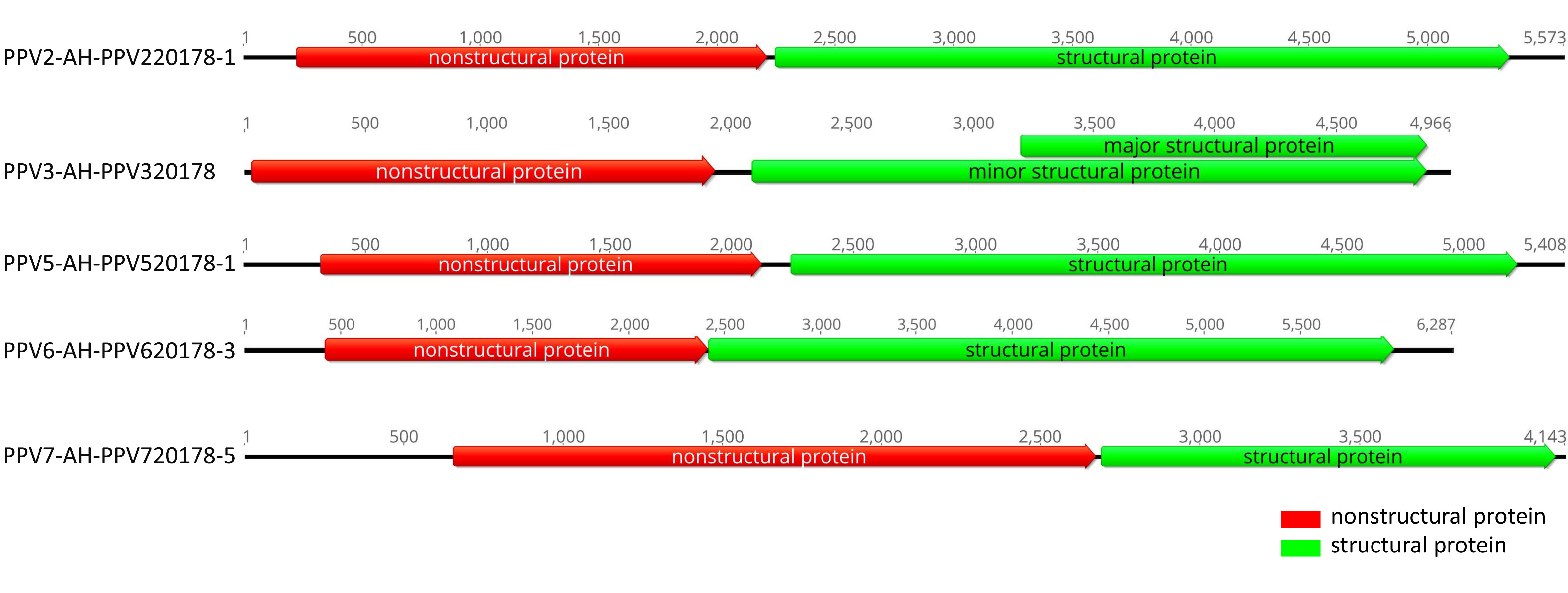

Supplement: Supplementary file 1 [file viruses-14-02048-s001.zip › Supplementary Figure S4.jpg]
